# Supplementary figures and images for: Immediate and Sustained Effects of Cobalt and Zinc-Containing Pigments on Macrophages
Source: Front Immunol. 2022 Jul 19;13:865239. doi: 10.3389/fimmu.2022.865239 (PMC9343594; doi:10.3389/fimmu.2022.865239)

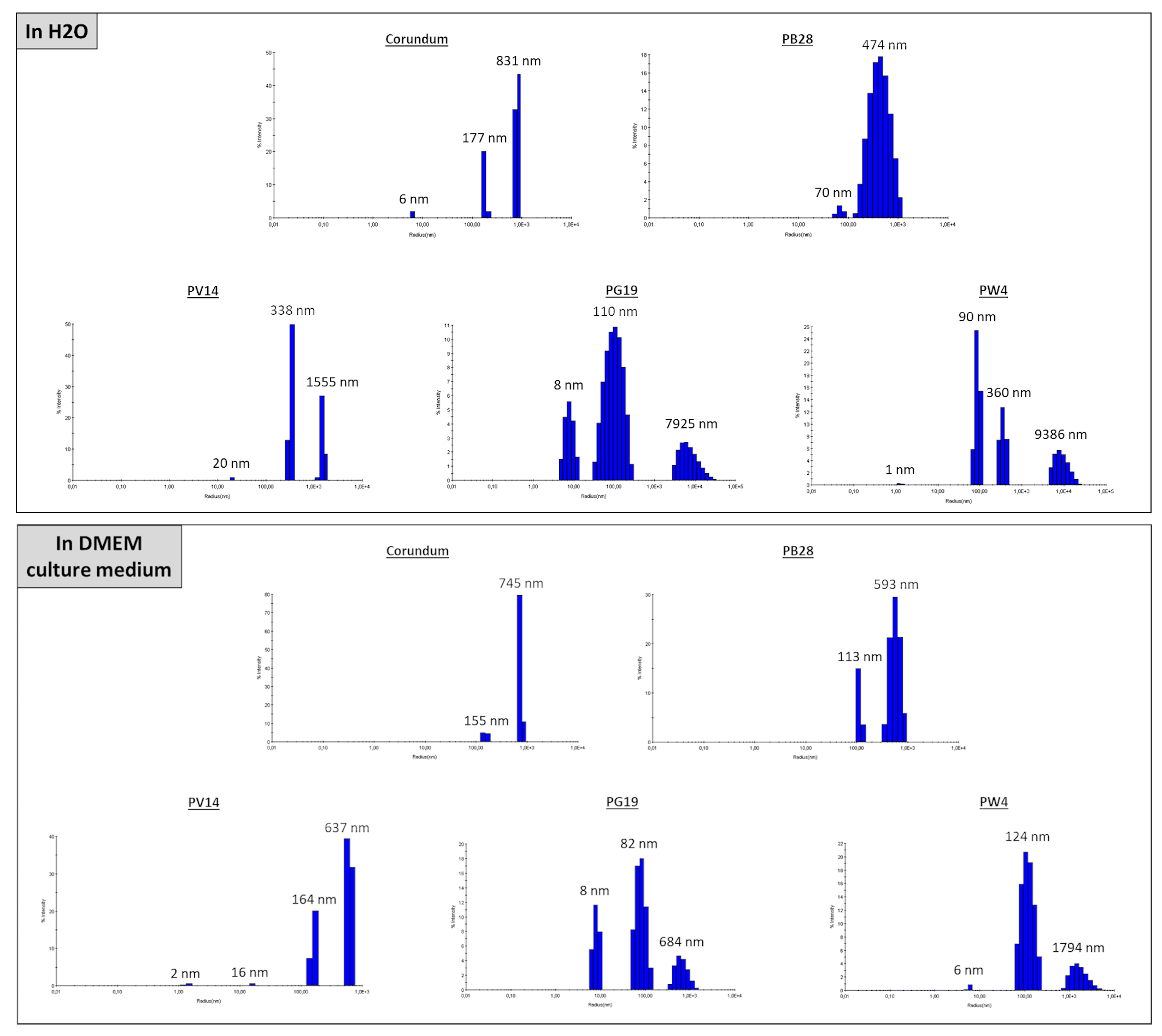

Supplement: Supplementary Data Sheet 1 — Example of hydrodynamic radius measurement (screen print of raw data) by DLS in H2O and DMEM culture medium. [file Image_1.jpeg]

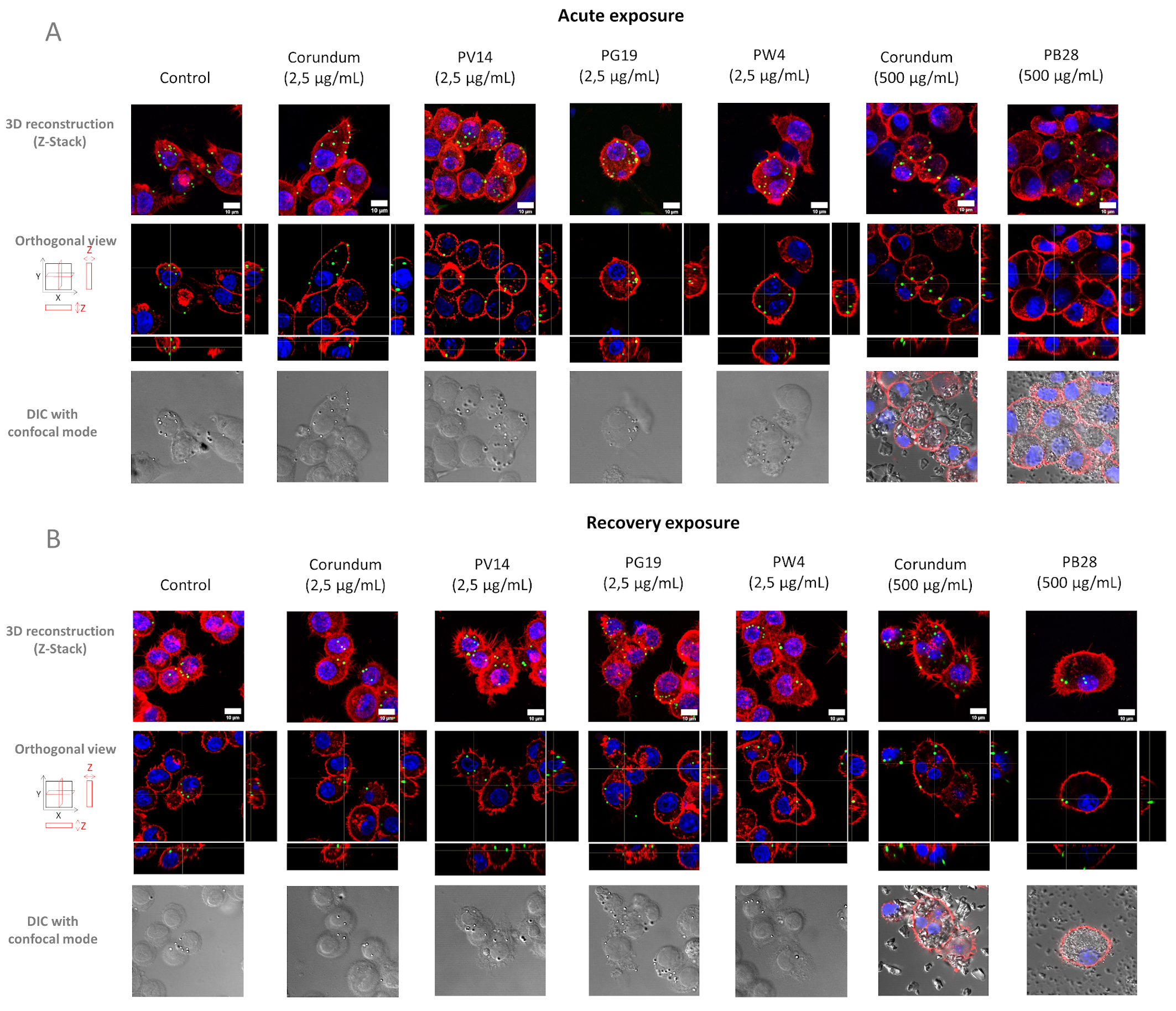

Supplement: Supplementary Data Sheet 2 — Visualization of phagocytic activity by confocal microscopy. Pannel A- Acute exposure of macrophages to pigments. Pannel B – End of recovery exposure of macrophages to pigment. Red color = Actin filament visualized with phalloidin-Atto 560. Blue color = Nucleus visualized with Dapi. Green color = Fluorescent yellow/green carboxylate- modified-polystyren beads (1 µm diameter). Grey color = differential interference contrast (DIC). Scale bar = 10 µm. [file Image_2.jpeg]

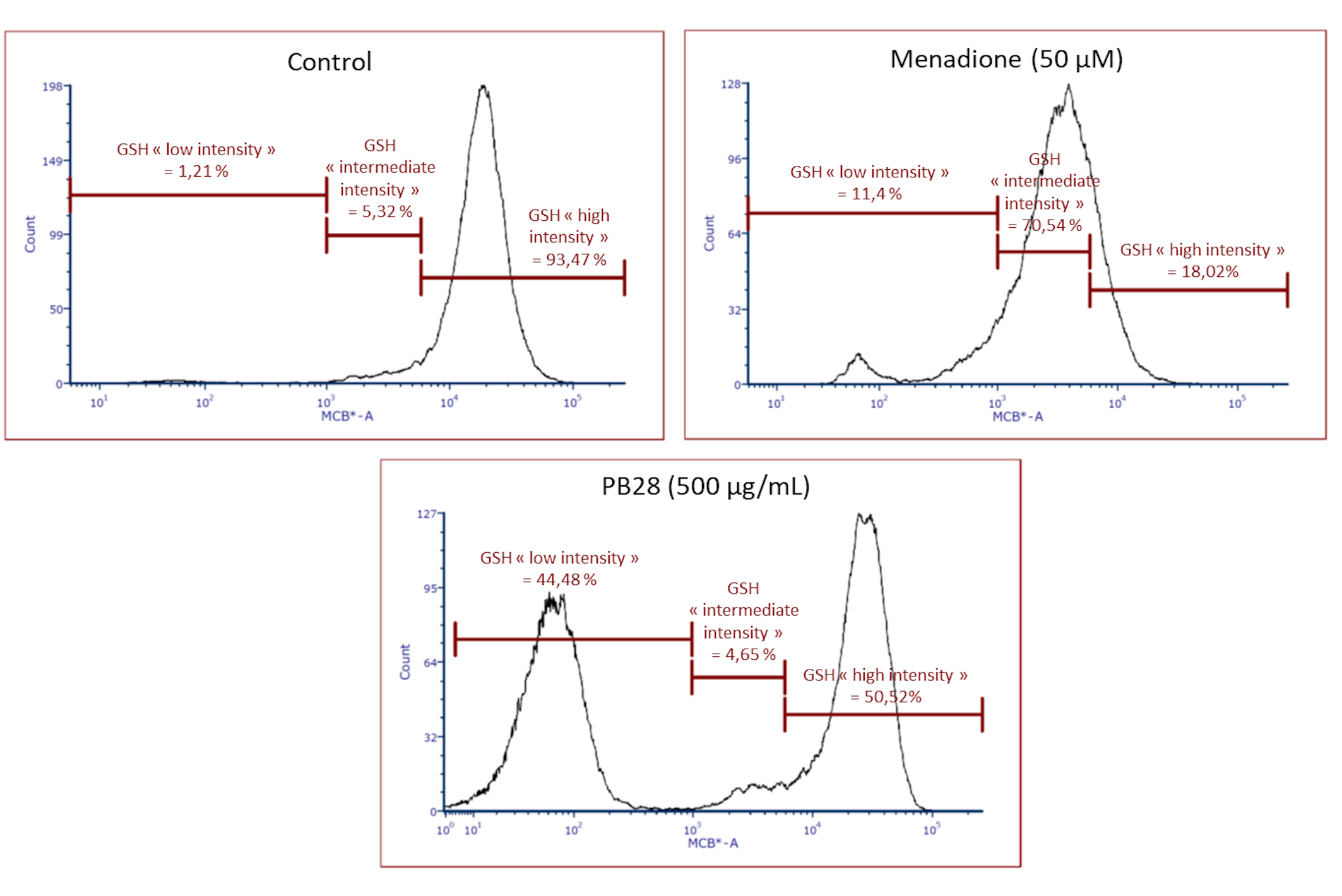

Supplement: Supplementary Data Sheet 3 — Examples of GSH results obtained via flow cytometry (Raw results of recovery exposure). [file Image_3.jpeg]

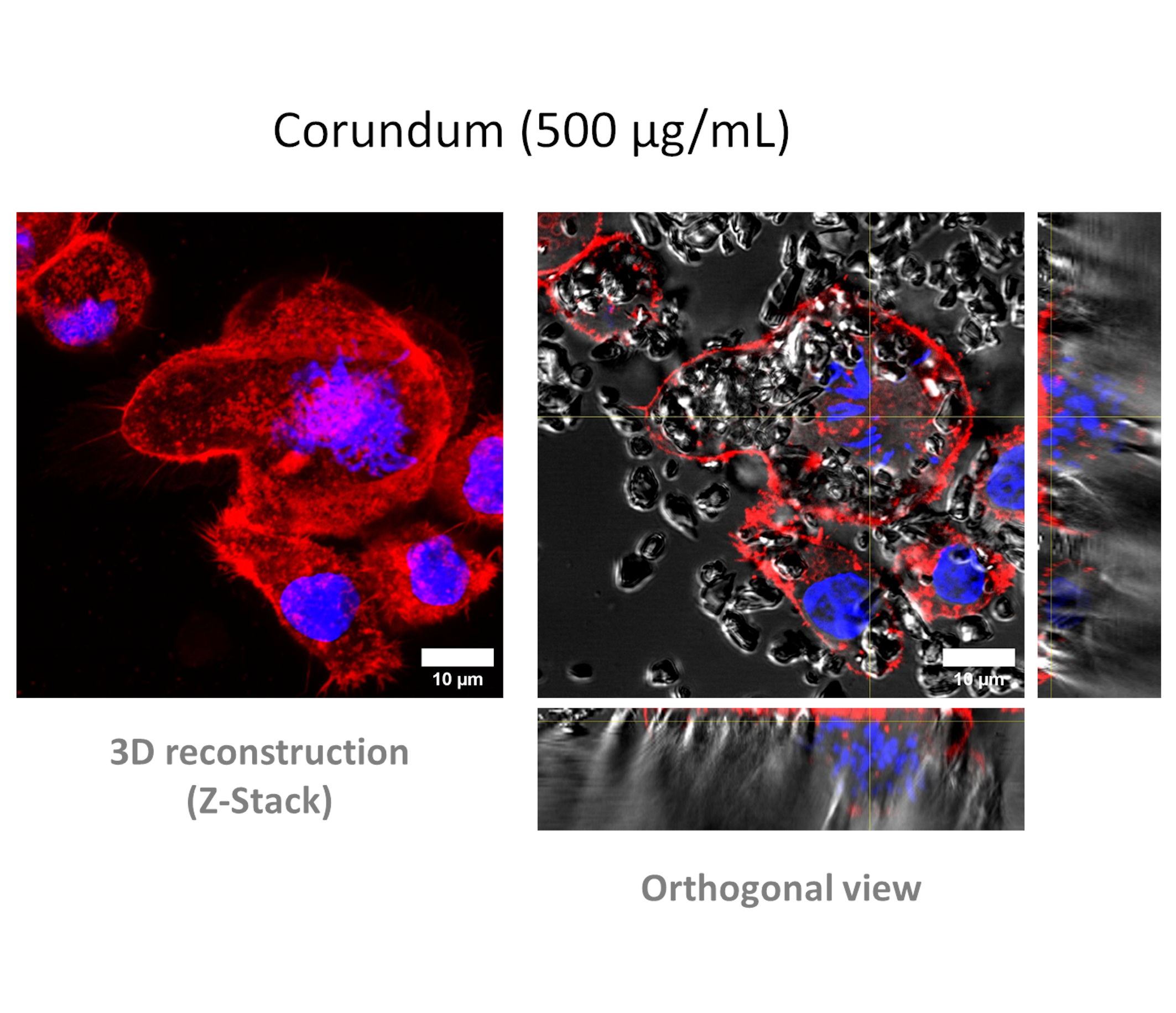

Supplement: Supplementary Data Sheet 4 — Confocal microscopy. Observation of actin conformation and nuclear integrity. Red = Actin (in red) stained with phalloidin-Atto 560. DNA (in blue) stained with Dapi. Grey = Corundum particles observed in confocal DIC. Scale bar = 10 µm. [file Image_4.jpeg]
